# Impact of Quantitatively Assessed Interstitial Lung Abnormalities on Long-Term Outcomes After Lung Cancer Surgery

**DOI:** 10.3390/jcm14165640

**Published:** 2025-08-09

**Authors:** Jae Hyun Jeon, Joonseok Lee, Jong Sun Park, In Kyu Park, Sowon Jang, Jung Woo Son, Woohyun Jung, Sukki Cho, Kwhanmien Kim

**Affiliations:** 1 Department of Thoracic and Cardiovascular Surgery, Seoul National University Bundang Hospital, Seoul National University College of Medicine, Seongnam 13620, Republic of Korea; fine1114@hanmail.net (J.H.J.); bnmbnmbnm1010@gmail.com (J.L.); minisjw@naver.com (J.W.S.); chucky0406@snubh.org (W.J.); skcho@snubh.org (S.C.); kmkim0070@snubh.org (K.K.); 2 Division of Pulmonary and Critical Care Medicine, Department of Internal Medicine, Seoul National University Bundang Hospital, Seoul National University College of Medicine, Seongnam 13620, Republic of Korea; parkjs@snubh.org; 3 Department of Thoracic and Cardiovascular Surgery, Seoul National University Hospital, Seoul National University College of Medicine, 101, Daehak-ro, Jongno-gu, Seoul 03080, Republic of Korea; 4 Department of Radiology, Seoul National University Bundang Hospital, Seoul National University College of Medicine, Seongnam 13620, Republic of Korea; swjang@snubh.org

**Keywords:** non-small-cell lung cancer, thoracic surgery, artificial intelligence, interstitial lung abnormality, survival

## Abstract

**Background**: This study evaluated the prognostic significance of quantitatively assessed interstitial lung abnormalities (ILAs) after lung cancer surgery. **Methods:** We included patients with pathologic stage I non-small-cell lung cancer (NSCLC) who underwent segmentectomy or lobectomy. ILAs were quantified using deep learning texture analysis software. Five-year overall survival (OS) was compared before and after propensity score matching. Competing risks for lung cancer and non-cancer mortality were also analyzed. **Results:** Among the 1711 patients, 263 (15.4%) comprised the ILA group. The ILA group was older and had a higher proportion of smokers and pathologic stage IB cases (all *p* < 0.001). The median follow-up period was 48.0 months. Before matching, 5-year OS was significantly worse in the ILA group than in the non-ILA group (82.5% vs. 93.4%, *p* < 0.001). After 2:1 matching (*N* = 697), 5-year OS remained lower in the ILA group (85.8% vs. 91.1%, *p* = 0.025). Multivariable Cox regression analysis showed that the presence of ILAs was associated with increased risk of all-cause mortality (HR 1.52, 95% CI 1.05–2.18, *p* = 0.025). Restricted cubic spline analysis revealed a nonlinear increase in mortality risk with greater fibrotic ILA burden. In competing risk analysis, death from lung cancer was similar between groups (2.9% vs. 4.2%, *p* = 0.3), whereas death from other causes was significantly higher in the ILA group (13.0% vs. 3.7%, *p* < 0.001). **Conclusions:** Quantitative assessment of ILAs may provide prognostic value in resected stage I NSCLC, particularly in patients with fibrotic changes.

## 1. Introduction

Interstitial lung disease refers to a group of disorders characterized by inflammation or fibrosis within the pulmonary interstitium and represents a well-established clinical diagnosis. In patients with non-small-cell lung cancer (NSCLC), the presence of ILD is widely recognized as being associated with increased postoperative complications and poorer long-term survival [1,2,3].

Interstitial lung abnormalities (ILAs) are incidental radiologic findings on chest computed tomography (CT), defined as non-dependent abnormalities affecting more than 5% of any lung zone in the absence of known or suspected interstitial lung disease [4]. With the increased use of CT imaging for cancer screening and routine health surveillance, these findings are increasingly detected, particularly among older adults and smokers. These radiologic findings have clinical relevance, being associated with a decline in pulmonary function, progression to interstitial lung disease, and increased risk of mortality [5,6,7]. Additionally, ILAs have been recognized as important in lung cancer patients, with implications for postoperative complications and long-term survival outcomes [8,9].

Despite growing awareness of their clinical importance, most prior studies have relied on visual assessment by radiologists, which is subjective and susceptible to interobserver variability. Recently, deep learning-based image analysis has emerged as a promising tool for objective and reproducible quantification of ILAs [10]. However, validation of quantitative assessments of ILAs in surgically resected lung cancer remains limited, especially in early-stage disease where subtle fibrotic changes may impact long-term outcomes [11,12,13].

In this study, we aimed to evaluate the prognostic impact of ILAs using AI-based quantitative analysis in patients with resected pathologic stage I NSCLC. We hypothesized that the presence of ILAs would be associated with worse long-term survival and an increased risk of non-cancer mortality after lung cancer surgery.

## 2. Material and Methods

This study was approved by the Institutional Review Board of Seoul National University Bundang Hospital (IRB number: B-2402-883-101, date: 1 February 2024), with a waiver of informed consent due to its retrospective design.

A total of 1872 patients who underwent segmentectomy or lobectomy with lymphadenectomy and were diagnosed with pathologic stage I non-small-cell lung cancer between 2010 and 2022 were identified from the institution’s prospectively maintained lung cancer database. Patients with a history of previous lung cancer treatment (*n* = 22), interstitial lung disease (*n* = 20), or receipt of any adjuvant therapy (chemotherapy, *n* = 117; radiotherapy, *n* = 2) were excluded. After exclusions, the medical records and radiologic images of 1711 patients were retrospectively reviewed for analysis.

### 2.1. Quantitative Assessment of ILAs Using AI-Based CT Analysis

**Figure 1:**
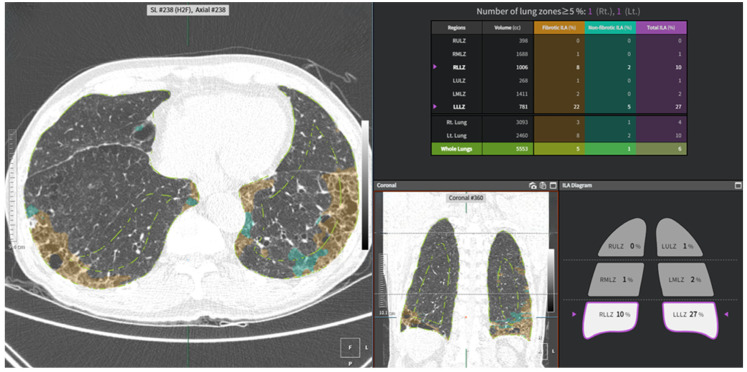
A user interface of an automated quantitative tool for interstitial lung abnormality (ILA). The system divides both lungs into upper, middle, and lower zones based on the levels of the inferior aortic arch and right inferior pulmonary vein (white dash line). Fibrotic ILA (orange), non-fibrotic ILA (mint), and total ILA (purple) extents are quantified in each zone and visualized on the CT images and in the summary table. Representative non-contrast chest CT images of a 75-year-old male smoker with subpleural fibrotic ILA (under the green line) demonstrate subpleural traction bronchiectasis with reticular opacities (orange) and ground-glass opacities (mint) in both lower lung zones.

ILAs were measured using commercially available AI-based software (AVIEW, version 1.2, Coreline Soft, Seoul, Republic of Korea). This fully automated program performed a pattern-based approach using a two-dimensional U-Net architecture to segment abnormal lung lesions on chest CT. The lung parenchyma was classified into three categories: (1) normal parenchyma; (2) fibrotic ILA components, including reticulation, honeycombing, and/or traction bronchiectasis; and (3) non-fibrotic ILA components, including ground-glass opacities. The performance of this program has been validated in recently published articles, demonstrating high concordance with radiologists and robust reproducibility [14,15]. The lungs were segmented into six anatomical zones based on anatomical landmarks such as the levels of the inferior aortic arch and the right inferior pulmonary vein, and fibrotic and non-fibrotic components were automatically quantified (Figure 1). ILA was defined as the sum of fibrotic and non-fibrotic components within each lung zone, representing non-dependent abnormalities involving at least 5% of any lung zone, according to the Fleischner Society guidelines [4].

### 2.2. CT Acquisition Protocols for Image Analysis

Chest CT scans used for analysis were obtained with either standard-dose or low-dose non-contrast protocols. CT images were reconstructed with a slice thickness of 1–1.25 mm and an interval of ≤1.25 mm. Specific acquisition parameters, such as tube voltage and current, were not standardized and varied according to scanner type and institutional practice. These acquisition parameters satisfied the technical requirements of the AVIEW software used for image analysis.

### 2.3. Data Collection

Baseline characteristics—including age, sex, smoking history, performance status, body mass index, preoperative pulmonary function, pathologic stage, tumor histology, and extent of surgery—were compared between the two groups. The pathologic stage was determined according to the eighth edition of the TNM classification for lung cancer [16]. All variables were obtained from a prospectively collected institutional database, and no missing values were present.

Postoperative surveillance was performed at 3- or 6-month intervals for the first 2 years, at 6- or 12-month intervals until 5 years postoperatively, and annually thereafter. The dates of last follow-up and death, along with the cause of death, if applicable, were recorded. For patients lost to follow-up, survival status was confirmed using death certificate data obtained from Statistics Korea.

### 2.4. Statistical Analysis

The primary outcome was 5-year overall survival (OS), defined as the interval from surgery to the most recent follow-up or death from any cause. Survival curves were generated using the Kaplan–Meier method and compared by log-rank test. Multivariable Cox proportional hazards models were used to adjust for confounding factors that are known to influence survival outcome, including age (continuous), sex (female vs. male), smoking history (never vs. ever), performance status (0–1 vs. ≥2), pulmonary function parameters (FEV1, FVC, and DLCO; all treated as continuous variables), extent of surgery (segmentectomy vs. lobectomy), pathologic stage (IA vs. IB), histology (adenocarcinoma vs. non-adenocarcinoma), and ILA group (no vs. yes). To improve model accuracy while minimizing complexity and the risk of overfitting, stepwise backward elimination based on the greatest reduction in Akaike information criterion was employed to identify the best-fitting subset of variables. Multicollinearity was assessed using variance inflation factors (VIFs). The proportional hazards assumption was evaluated using Schoenfeld residuals, and the model’s discriminative ability was assessed using Harrell’s C-index. Hazard ratios (HRs) and 95% confidence intervals (CIs) were calculated for each covariate. Restricted cubic spline analysis was additionally performed to evaluate dose–response patterns and to identify potential threshold effects of ILA components [17].

To minimize selection bias, propensity score-matched analysis was performed. The propensity score was estimated using a logistic regression model that included age, sex, smoking history, performance status, pulmonary function tests, extent of surgery, histology, and pathologic stage. Patients were matched in a 2:1 ratio using the nearest-neighbor method with a caliper width of 0.1. Covariate balance before and after matching was assessed using the standardized mean difference (SMD), with SMD > 0.1 indicating a meaningful imbalance [18].

The cumulative incidence of death from primary lung cancer and from other causes was also estimated using competing risks analysis. Death from primary lung cancer was defined as death from cancer progression, including locoregional or distant progression. All other causes of death, including non-cancer and unknown origins, were considered competing events. Differences in cumulative incidences were assessed using Gray’s test and the Fine–Gray model. Variables with *p* < 0.10 in univariable analyses were included in the multivariable competing risk regression models to identify independent predictors of cause-specific mortality.

Continuous variables were summarized as medians with interquartile ranges, and categorical variables as total numbers with percentages. Differences between the two groups were assessed using the Kruskal–Wallis test for continuous variables and either the Pearson chi-square or Fisher’s exact test for categorical variables. The Fisher exact test was applied when ≥20% of the cells in a contingency table had expected values < 5. All *p*-values less than 0.05 were considered statistically significant. Statistical analysis was performed using R statistical software (version 4.1.0; R Foundation for Statistical Computing, Vienna, Austria).

## 3. Results

### 3.1. Clinical and Pathological Characteristics

Of the 1711 patients, 263 (15.4%) were classified into the ILA group. Compared to the non-ILA group, patients in the ILA group were older (71.0 vs. 65.0 years, *p* < 0.001), had a higher proportion of ever-smokers (54.8% vs. 45.5%, *p* < 0.001), and included more patients with performance status ≥2 (14.4% vs. 5.9%, *p* < 0.001). The ILA group also had a significantly lower median diffusion capacity (94.0% vs. 102.0%, *p* < 0.001). Regarding oncologic features, the ILA group showed a higher prevalence of pathologic stage IB (34.2% vs. 17.6%, *p* < 0.001) and non-adenocarcinoma histology (26.6% vs. 11.4%, *p* < 0.001) (Table 1).

### 3.2. Survival Outcomes in the Overall Study Cohort

**Figure 2:**
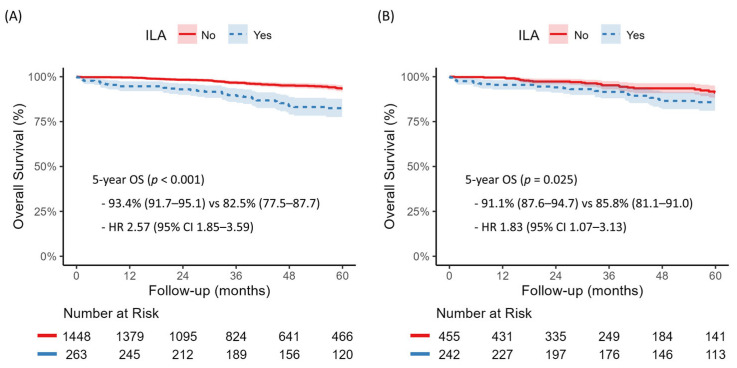
Kaplan–Meier curves for 5-year overall survival according to the presence of interstitial lung abnormalities (ILA) in patients with stage I invasive non-small-cell lung cancer who underwent segmentectomy or lobectomy. (**A**) Overall study cohort (*N* = 1711). (**B**) Propensity score-matched (2:1) cohort (*N =* 697).

The median follow-up duration in the entire cohort was 48.0 months (interquartile range, 24.8–73.5 months). Five-year OS was 93.4% (95% CI, 91.7–95.1%) in the non-ILA group and 82.5% (77.5–87.7%) in the ILA group (HR 2.57, 95% CI 1.85–3.59, *p* < 0.001) (Figure 2A). In multivariable Cox regression analysis, ILA remained an independent predictor of all-cause mortality (HR 1.50, 95% CI 1.04–2.15, *p* = 0.029) (Table 2). Model diagnostics showed no violation of the proportional hazard assumption (global *p* = 0.777), no significant multicollinearity (all VIFs < 2.5, with the highest being 2.29 for FEV1), and good discriminative ability (Harrell’s C-index = 0.792).

**Figure 3:**
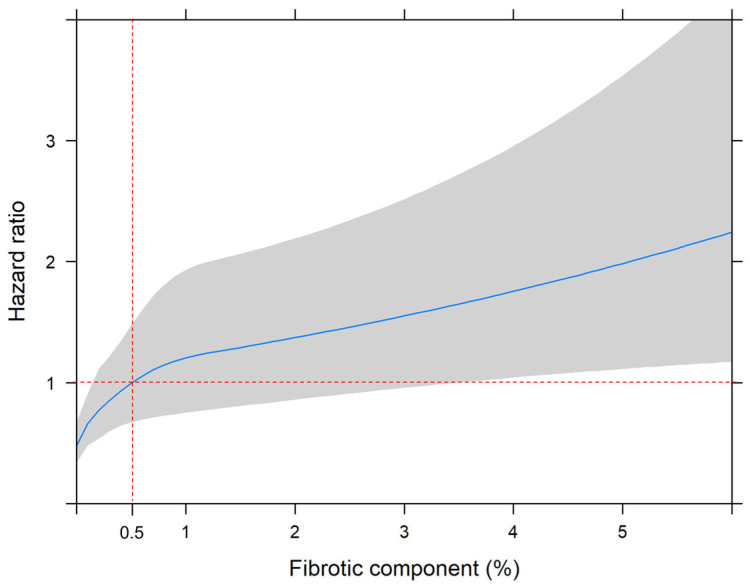
Nonlinear association between fibrotic components of ILAs and overall survival using restricted cubic spline analysis. The blue line represents the estimated hazard ratio according to the fibrotic components (%), with gray areas indicating the 95% confidence interval. A threshold effect is observed around a 0.5% proportion of fibrotic ILAs (red-dashed line) relative to the total lung volume, beyond which the hazard ratio increases progressively.

**Figure 4:**
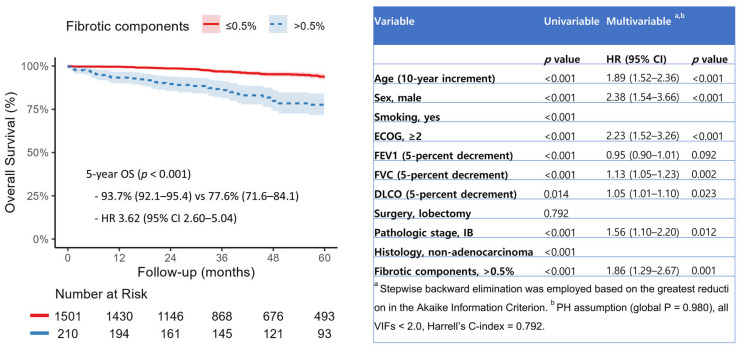
Kaplan–Meier survival curves and multivariable Cox regression analysis according to the fibrotic components of interstitial lung abnormalities (ILA), dichotomized at a 0.5% threshold.

When fibrotic and non-fibrotic components were entered as continuous variables in the multivariable model, only fibrotic components were significantly associated with increased all-cause mortality (HR 1.19, 95% CI 1.09–1.30, *p* < 0.001) (Supplementary Table S1). Restricted cubic spline analysis revealed a nonlinear increase in mortality risk with rising fibrotic ILA burden, with a threshold effect observed at approximately 0.5% of total lung volume (Figure 3). When dichotomized at this threshold, patients with fibrotic ILAs > 0.5% exhibited significantly worse overall survival (HR 3.62, 95% CI 2.60–5.04, *p* < 0.001), with an adjusted hazard ratio of 1.86 (95% CI, 1.29–2.67; *p* = 0.001) (Figure 4).

### 3.3. Survival Outcomes in Matched Cohort

After propensity score matching, 697 patients were included. Baseline characteristics were well balanced between the two groups, with all SMDs < 0.1 (Table 3). The covariate balance before and after matching is further illustrated in the Love plot (Supplementary Figure S1). The median follow-up duration was 49.6 months (interquartile range, 25.3–79.0). Kaplan–Meier curves demonstrated that 5-year OS remained significantly lower in the ILA group compared to the non-ILA group (85.8% vs. 91.4%; HR 1.83, 95% CI 1.07–3.13, *p* = 0.025) (Figure 2B). Sensitivity analyses were conducted using alternative matching strategies, which remained consistent outcomes in both 1:3 matching with a caliper of 0.1 (HR 1.84, 95% CI 1.12–3.02, *p* = 0.014) and 1:2 matching with a caliper of 0.05 (HR 1.84, 95% CI 1.09–3.11, *p* = 0.022) (Supplementary Figure S2).

### 3.4. Competing Risk Analysis for Death from Lung Cancer and Other Causes

**Figure 5:**
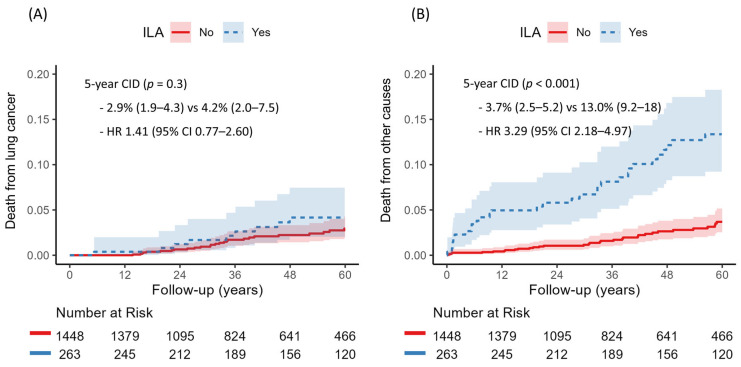
Cumulative incidence of (**A**) death from primary lung cancer and (**B**) death from other causes according to the presence of interstitial lung abnormalities (ILA).

There were 150 deaths in total, including 59 deaths from primary lung cancer and 91 from other causes. The cumulative incidence of death from other causes was significantly higher in the ILA group (13.0% vs. 3.7%, *p* < 0.001), while lung cancer–related death did not differ significantly between groups (4.2% vs. 2.9%, *p* = 0.30) (Figure 5). Multivariable competing risk regression analysis indicated that the presence of ILAs was independently associated with increased risk of non-cancer death (HR 1.76, 95% CI 1.12–2.76, *p* = 0.014) (Table 4).

## 4. Discussion

In this retrospective cohort of patients with resected stage I NSCLC, AI-based quantitative analysis demonstrated that the presence of ILAs was associated with worse long-term survival outcomes. After adjusting for clinical and pathological factors, ILAs remained an independent predictor of all-cause mortality in both multivariable Cox proportional hazards and propensity score–matched analyses. Additionally, restricted cubic spline analysis revealed a nonlinear association between quantitatively measured fibrotic burden and mortality risk. Competing risk analysis further showed that ILAs were significantly associated with increased risk of non-cancer death.

ILAs have been recognized as prognostic indicators in various clinical settings, including early-stage lung cancer. However, they are often under-reported or misclassified, particularly in borderline or mild cases, due to substantial interobserver variability in visual assessment [15,19]. Recently, deep learning-based CT texture analysis tools have emerged as promising alternatives, offering objective and reproducible results [20]. These tools enable precise measurement of ILA burden and support dose–response modeling rather than simple binary classification, thus facilitating more nuanced risk stratification [21]. However, few studies have examined AI-based ILA quantification in resectable lung cancer, and the available evidence is limited by heterogeneous study cohorts and relatively small sample sizes [11,12].

This study has several methodological strengths. First, we analyzed a relatively large and homogeneous cohort of 1711 patients with stage I NSCLC who underwent curative resection, using a prospective institutional lung cancer database. To minimize bias from incomplete follow-up, mortality status and cause of death were supplemented using national registry data. Second, ILAs were assessed with a well-validated, fully automated AI-based software platform, enabling objective and reproducible quantification across the cohort. Third, multiple complementary statistical methods—including multivariable Cox regression, propensity score matching, and competing risk analysis—were used to adjust for potential confounders and enhance the reliability of our findings. Despite the inherent limitations of these approaches, quantitative assessment of ILAs was an independent predictor of worse overall survival and higher risk of non-cancer mortality.

Fibrotic ILAs, characterized by architectural distortion with traction bronchiectasis or honeycombing, have been associated with higher rates of radiologic progression and all-cause mortality [22,23]. Prior studies have relied on subjective radiologist analysis, whereas we quantified both fibrotic and non-fibrotic ILA components and evaluated their associations with clinical outcomes. Restricted cubic spline modeling identified a nonlinear increase in mortality risk with rising fibrotic ILA burden, with a threshold effect at approximately 0.5% of total lung volume. These findings suggest that preoperative quantification may help identify high-risk patients and inform decisions regarding surgical extent and postoperative surveillance. However, as this outcome is based on a single-center exploratory analysis, external validation is required to confirm the generalizability and predictive value of the proposed threshold.

This study has several limitations. First, it was a retrospective observational study conducted at a single institution, without a comprehensive, prospectively designed protocol. Second, despite efforts to minimize selection bias using multivariable regression and propensity score matching, residual confounding may still influence clinical outcomes. In particular, due to the heterogeneous nature of deaths other than lung cancer, further subclassification was not performed, which may have influenced the results of competing risk analyses. Third, although ILA quantification was performed with fully automated AI-based software, potential inaccuracies in image segmentation or pattern classification may persist. Additionally, variability in CT acquisition protocols across patients may affect the consistency of ILA quantification. These limitations cannot be addressed by a single-center study; therefore, well-designed, randomized, multicenter prospective trials are needed to establish more robust scientific evidence.

## 5. Conclusions

Quantitative assessment of ILAs may have prognostic significance for long-term survival in resected stage I NSCLC, particularly in cases with fibrotic features. While further validation is required, incorporating ILA analysis into routine preoperative evaluation may improve risk stratification and help guide surgical planning. 

## Figures and Tables

**Table 1:** Clinical and pathological characteristics of the overall study cohort (*N* = 1711).

	Total (*N* = 1711)	Non-ILA Group ^a^ (*N* = 1448)	ILA Group ^a^ (*N* = 263)	*p*-Value
Age, years	66.0 (59.0–73.0)	65.0 (59.0–72.0)	71.0 (65.0–77.0)	<0.001
Sex, male	839 (49.0)	693 (47.9)	146 (55.5)	0.027
Smoking, yes	803 (46.9)	659 (45.5)	144 (54.8)	0.007
ECOG PS, ≥2	124 (7.2)	86 (5.9)	38 (14.4)	<0.001
FEV1, %	104.0 (92.0–116.0)	104.0 (93.0–116.0)	102.0 (86.0–115.0)	0.029
FVC, %	101.0 (92.0–111.0)	101.0 (92.0–111.0)	100.0 (88.0–110.0)	0.014
DLCO, %	101.0 (89.0–113.0)	102.0 (91.0–114.0)	94.0 (81.5–108.0)	<0.001
Surgery, lobectomy	1504 (87.9)	1269 (87.6)	235 (89.4)	0.495
Pathologic stage, IB	345 (20.2)	255 (17.6)	90 (34.2)	<0.001
Histology, adenocarcinoma	1476 (86.3)	1283 (88.6)	193 (73.4)	<0.001
Fibrotic component, %	0.1 (0.0–0.2)	0.1 (0.0–0.1)	0.7 (0.3–1.6)	<0.001
Non-fibrotic component, %	0.2 (0.1–0.4)	0.1 (0.1–0.2)	1.8 (0.8–3.3)	<0.001

^a^ Median (interquartile ranges); number (%) DLCO, diffusion capacity of the lung for carbon monoxide; ECOG PS, Eastern Cooperative Oncology Group performance status; FEV1, forced expiratory volume in 1 s; FVC, forced vital capacity; ILA, interstitial lung abnormalities.

**Table 2:** Stepwise multivariable Cox proportional-hazard model for overall survival.

Variable	Univariable	Multivariable ^a,b^	
	*p*-Value	HR (95% CI)	*p*-Value
Age (10-year increment)	<0.001	1.88 (1.50–2.34)	<0.001
Sex, male	<0.001	2.47 (1.59–3.83)	<0.001
Smoking, yes	<0.001		
ECOG, ≥2	<0.001	2.27 (1.55–3.33)	<0.001
FEV1 (5-percent decrement)	<0.001	0.95 (0.89–1.01)	0.084
FVC (5-percent decrement)	<0.001	1.13 (1.05–1.23)	0.002
DLCO (5-percent decrement)	0.014	1.05 (1.01–1.10)	0.02
Surgery, lobectomy	0.792		
Pathologic stage, IB	<0.001	1.52 (1.08–2.14)	0.018
Histology, non-adenocarcinoma	<0.001	1.32 (0.90–1.92)	0.152
ILA group, yes	<0.001	1.50 (1.04–2.15)	0.029

^a^ Stepwise backward elimination was employed to select the best-fitting subset of variables for the multivariable model, based on the greatest reduction in the Akaike information criterion. ^b^ PH assumption (global *p* = 0.777), all VIFs < 2.5, Harrell’s C-index = 0.792. CI, confidence interval; DLCO, diffusion capacity of the lung for carbon monoxide; ECOG PS, Eastern Cooperative Oncology Group performance status; FEV1, forced expiratory volume in 1 s; FVC, forced vital capacity; HR, hazard ratio; ILA, interstitial lung abnormalities.

**Table 3:** Clinical and pathological characteristics of the matched cohorts (*N* = 697).

	Non-ILA Group ^a^ (*N* = 455)	ILA Group ^a^ (*N* = 242)	*p*-Value	SMD
Age, years	70.0 (64.0–75.0)	70.0 (64.0–76.0)	0.460	0.051
Sex, male	233 (51.2)	131 (54.1)	0.512	0.059
Smoking, yes	230 (50.5)	128 (52.9)	0.610	0.047
ECOG, ≥2	54 (11.9)	28 (11.6)	>0.999	0.009
FEV1, %	105.0 (91.0–117.0)	103.0 (87.0–115.0)	0.221	0.061
FVC, %	101.0 (92.0–110.5)	100.0 (88.0–110.0)	0.263	0.060
DLCO, %	97.0 (86.0–108.0)	95.0 (84.0–108.0)	0.510	0.074
Surgery, lobectomy	408 (89.7)	217 (89.7)	>0.999	<0.001
Pathologic stage, IB	137 (30.1)	75 (31.0)	0.877	0.019
Histology, adenocarcinoma	358 (78.7)	186 (76.9)	0.648	0.044
Fibrotic component, %	0.1 (0.0–0.2)	0.6 (0.3–1.5)	<0.001	0.969
Non-fibrotic component, %	0.2 (0.1–0.3)	1.8 (0.8–3.1)	<0.001	0.885

^a^ Median (interquartile ranges); number (%) DLCO, diffusion capacity of the lung for carbon monoxide; ECOG PS, Eastern Cooperative Oncology Group performance status; FEV1, forced expiratory volume in 1 s; FVC, forced vital capacity; ILA, interstitial lung abnormalities.

**Table 4:** Stepwise multivariable competing risk hazard model for death from other causes.

Variables	Univariable	Multivariable ^a^	
	*p*-Value	HR (95% CI)	*p*-Value
Age (10-year increment)	<0.001	2.04 (1.53–2.72)	<0.001
Sex, male	<0.001	3.06 (1.66–5.64)	<0.001
Smoking, yes	<0.001		
ECOG, ≥2	<0.001	2.40 (1.44–3.99)	0.001
FEV1 (5-percent decrement)	<0.001	0.93 (0.83–1.04)	0.184
FVC (5-percent decrement)	<0.001	1.16 (1.02–1.33)	0.026
DLCO (5-percent decrement)	0.014	1.09 (1.02–1.17)	0.012
Surgery, lobectomy	0.792		
Pathologic stage, IB	<0.001	1.43 (0.90–2.29)	0.126
Histology, non-adenocarcinoma	<0.001		
ILA group, yes	<0.001	1.76 (1.12–2.78)	0.013

^a^ Stepwise backward elimination was employed to select the best-fitting subset of variables for the multivariable model, based on the greatest reduction in the Akaike information criterion. CI, confidence interval; DLCO, diffusion capacity of the lung for carbon monoxide; ECOG PS, Eastern Cooperative Oncology Group performance status; FEV1, forced expiratory volume in 1 s; FVC, forced vital capacity; HR, hazard ratio; ILA, interstitial lung abnormalities.

## Data Availability

The datasets used and/or analyzed during the current study are available from the corresponding author upon reasonable request.

## Supplementary Materials

The following supporting information can be downloaded at: https://www.mdpi.com/article/10.3390/jcm14165640/s1, Figure S1: Love plot of covariate balance before and after propensity score matching (*N* = 697). Figure S2: Sensitivity analyses using alternative propensity score matching strategies. (A) 1:3 matching with caliper = 0.1 (*N* = 885). (B) 1:2 matching with caliper = 0.2 (*N* = 710). Table S1: Stepwise multivariable Cox proportional-hazard model for overall survival, including the percentage of fibrotic and non-fibrotic components.
