# Supplementary material for: Impact of Quantitatively Assessed Interstitial Lung Abnormalities on Long-Term Outcomes After Lung Cancer Surgery
Source: J Clin Med. 2025 Aug 9;14(16):5640. doi: 10.3390/jcm14165640 (PMC12386349; doi:10.3390/jcm14165640)

Supplementary Table S1. Stepwise multivariable Cox proportional-hazard model for overall survival including the percentage of fibrotic and non-fibrotic components.

**Variable**	**Univariable**	**Multivariable** [a,b]	
	***p* value**	**HR (95% CI)**	***p* value**
Age (10-year increment)	<0.001	1.94 (1.55–2.42)	<0.001
Sex, male	<0.001	2.16 (1.44–3.24)	<0.001
Smoking, yes	<0.001		
ECOG, >=2	<0.001	2.01 (1.38–2.93)	<0.001
FEV1 (5-percent decrement)	<0.001		
FVC (5-percent decrement)	<0.001	1.08 (1.02–1.14)	0.012
DLCO (5-percent decrement)	0.014	1.04 (0.99–1.08)	0.112
Surgery, lobectomy	0.792		
Pathologic stage, IB	<0.001	1.55 (1.10–2.20)	0.012
Histology, non-adenocarcinoma	<0.001		
Fibrotic components (1-percent increment)	<0.001	1.19 (1.09–1.30)	<0.001
Non-fibrotic components (1-percent increment)	0.124		

[a] Stepwise backward elimination was employed to select best-fitting subset of variables for the multivariable model, based on the greatest reduction in the Akaike Information Criterion. [b] PH assumption (global P = 0.960), all VIFs < 2.0, Harrell's C-index = 0.797. CI, confidence interval; DLCO, diffusion capacity of the lung for carbon monoxide; ECOG PS, Eastern Cooperative Oncology Group performance status, FEV1 forced expiratory volume in 1 second; FVC, forced vital capacity; HR, hazard ratio; ILA, interstitial lung abnormalities.

Supplementary Figure S1: Love plot of covariate balance before and after propensity score matching (N = 697).

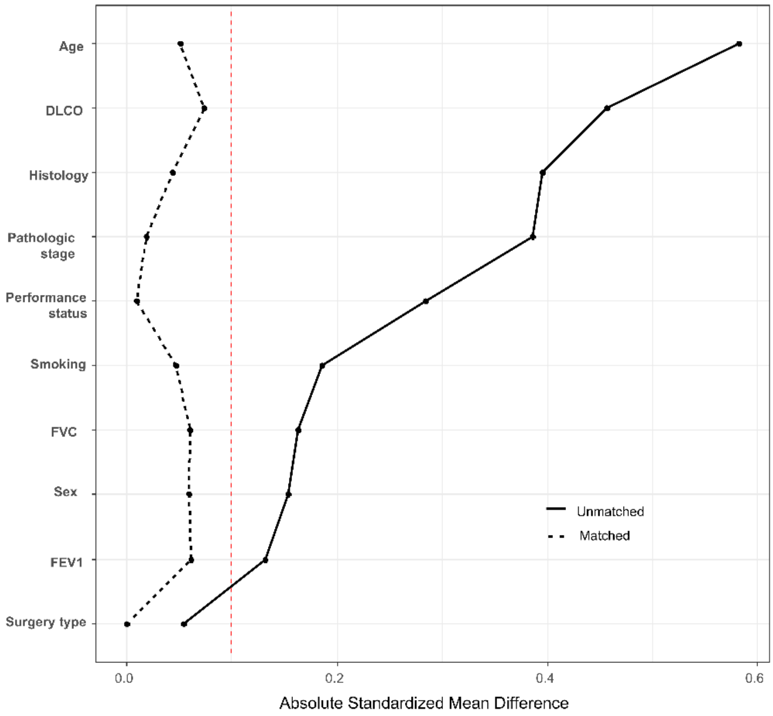

Supplementary Figure S2: Sensitivity analyses using alternative propensity score matching strategies. (A) 1:3 matching with caliper = 0.1 (*N* = 885). (B) 1:2 matching with caliper = 0.2 (*N* = 710).

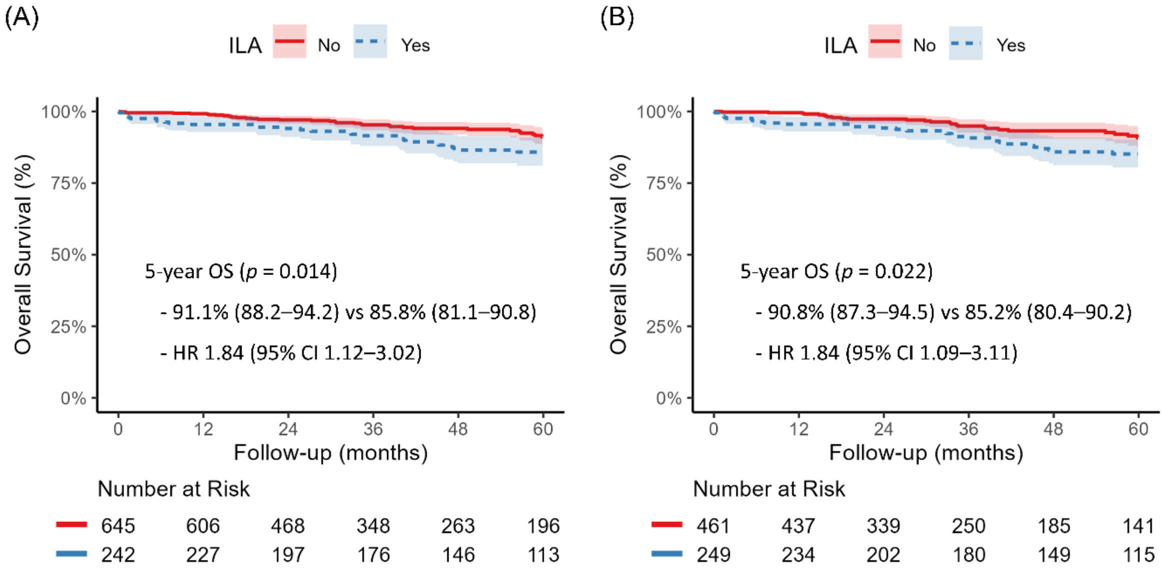

Supplement: Supplementary file 1 [file jcm-14-05640-s001.zip › jcm-3773871-supplementary.pdf]
